# Persistent pulmonary hypertension in children after apparent resolution of ultrasound-defined pulmonary hypertension associated with bronchopulmonary dysplasia

**DOI:** 10.1007/s00431-024-05843-6

**Published:** 2024-11-19

**Authors:** Mami Takeoka, Hirofumi Sawada, Yoshihide Mitani, Hiroyuki Ohashi, Noriko Yodoya, Kazunobu Ohya, Naoki Tsuboya, Tomoya Harada, Masahiro Hirayama

**Affiliations:** Department of Pediatrics, Mie University Graduate School of Medicine, 2-174 Edobashi, Tsu Mie, 514-8507 Japan

**Keywords:** Bronchopulmonary dysplasia, Pulmonary hypertension, Extremely premature infants, Cardiac catheterization

## Abstract

To evaluate pulmonary hemodynamics in patients who had been followed up for bronchopulmonary dysplasia (BPD)-associated pulmonary hypertension (PH) in the mid-term by right heart catheterization (RHC). A retrospective study was conducted among 56 patients who were born at a gestational age < 28 weeks during 2018–2020, and the consecutive patients complicated by BPD and ultrasound-defined PH (*n* = 10, 18%), of whom 7 patients were treated with sildenafil, were examined by RHC in the mid-term follow-up (median age: 25 months [IQR: 19–32]). Ultrasound study at RHC showed improvement in PH as represented by left ventricular end-systolic eccentricity index (median [IQR], 1.05 [IQR: 1.03–1.06] vs. 1.18 [IQR: 1.15–1.33]), pulmonary artery acceleration time (PAAT) (mean ± SD, 97.3 ± 18.2 vs. 55.2 ± 10.1) and a ratio of PAAT to right ventricular ejection time (0.35 ± 0.05 vs. 0.25 ± 0.04) compared with those at 40 weeks of postmenstrual age (PMA) (*p* < 0.05, respectively). RHC (mean pulmonary artery [PA] pressure: 21 [19–22] mmHg; pulmonary vascular resistance index [PVRi]: 2.63 [1.95–2.94] Wood Units·m^2^) revealed that 5 infants (50%) had mild PH (21–24 mmHg) and reduced peripheral PAs by pulmonary wedge angiography. The presence of emphysema on chest CT at 40 weeks of PMA and moderate/marked reduction of contrast filling in peripheral PAs on angiography were correlated with increased PVRi (*p* < 0.05, respectively).

*Conclusion*: We observed that even after apparent resolution of ultrasound-defined PH, half of patients had catheterization-defined PH and vasculopathy at 2 years of age. These findings warrant long-term follow-up studies of BPD-associated PH.

**Table:** 

**What is Known:** *• Pulmonary hypertension (PH) is associated with increased mortality in infants with bronchopulmonary dysplasia (BPD). Mortality in patients with BPD-associated PH predominantly occurs within the first 6 months of life and most patients who survive beyond 6 months show resolution of PH on echocardiography by 2 to 3 years of age.* **What is New:** *• Even after the resolution of echocardiography-derived PH in infants with BPD-associated PH in the mid-term, the majority of such patients have abnormal pulmonary vasculature with PH or mildly elevated pulmonary artery pressure on right heart catheterization (RHC), indicating that echocardiography is insufficient and RHC may be necessary to follow up this population of patients*.

**Supplementary Information:**

The online version contains supplementary material available at 10.1007/s00431-024-05843-6.

## Introduction

Pulmonary hypertension (PH) is a serious complication of bronchopulmonary dysplasia (BPD) in premature infants, which is characterized by impaired alveolarization and abnormal vascular development [1]. Severe cases have high mortality within half a year after 36 weeks of postmenstrual age (PMA) and a majority of BPD-associated PH (BPD-PH) patients after such period have an apparent resolution of PH on echocardiography [2, 3]. Although PH was recently defined as a mean pulmonary artery pressure (mPAP) > 20 mmHg by catheterization [4], it is unknown whether catheterization-defined PH persists in these patients in the mid-term. We therefore investigated PH and vasculopathy in infants who had been followed up for BPD-PH in the mid-term by right heart catheterization (RHC) and angiography.

## Methods

This is a retrospective observational study of consecutive patients (*n* = 10), who were born at GA < 28 weeks, diagnosed with BPD-PH at 40 weeks of PMA during 2018–2020 and evaluated by RHC and pulmonary wedge angiography under general anesthesia in the mid-term follow-up at Mie University Hospital, the only tertiary referral center for Pediatric Cardiology in a tertiary medical service area Mie prefecture, Japan. This study was approved by the Clinical Research Ethics Review Committee of Mie University Hospital (H2019-039) and was conducted in accordance with the Declaration of Helsinki. Included were patients who had an echocardiographic diagnosis of PH at 40 weeks of PMA, whose parents gave informed consent for RHC to monitor pulmonary hemodynamics after initiating PH-specific therapy or for hemodynamic evaluation of congenital heart disease (CHD). Patients with congenital malformation syndrome and patients who did not give informed consent or lost to follow up were excluded. Ultrasound PH screening was performed in an unsedated state at 40 weeks of PMA for all infants (*n *= 56) born at a GA < 28 weeks in a hospital affiliated with Mie University hospital. Ultrasound-derived PH parameters included were tricuspid regurgitation velocity > 2.5 m/s, left ventricular end-systolic eccentricity index (sEI) > 1.1, pulmonary artery acceleration time (PAAT) < 90 ms or a ratio of PAAT to right ventricular ejection time (PAAT/RVET) < 0.31 in accordance with previous reports including a guideline [4–6]. Sildenafil was initiated in patients with ultrasound-defined PH at a dose of 0.5 mg/kg/dose t.i.d. in NICU if desaturation while crying or the presence of risk factors including > 60 days exposure to mechanical ventilation, oligohydramnios, small for gestational age, sepsis, and moderate-severe BPD [7] were found. Severe BPD at 36 weeks of PMA was defined in accordance with the National Institute of Child Health and Human Development classification [8]. Severe emphysema was defined by chest CT findings as multiple bullae and blebs > 5 mm in diameter [9]. Pulmonary vascular hypoplasia was classified as normal, mild, moderate, or marked according to the degree of contrast filling of PAs in pulmonary wedge angiography [10], which was determined by three pediatric cardiologists.

Values are reported as number and percentage of values for categorical data, mean ± standard deviation or median (interquartile range [IQR]) for continuous variables as appropriate. Comparisons between the groups were made using the Fisher’s exact test for categorical data, paired *t*-test for the data that were normally distributed, Wilcoxon signed-rank test or Mann–Whitney *U* test for the variables that were not normally distributed. Missing data were excluded from all analyses. Pulmonary and systemic blood flows were calculated with the Fick method with oxygen consumption estimated by the method of LaFarge and Miettinen. The analysis was performed using GraphPad Prism version 10.0. A p value < 0.05 was considered statistically significant.

## Results

**Fig. 1:**
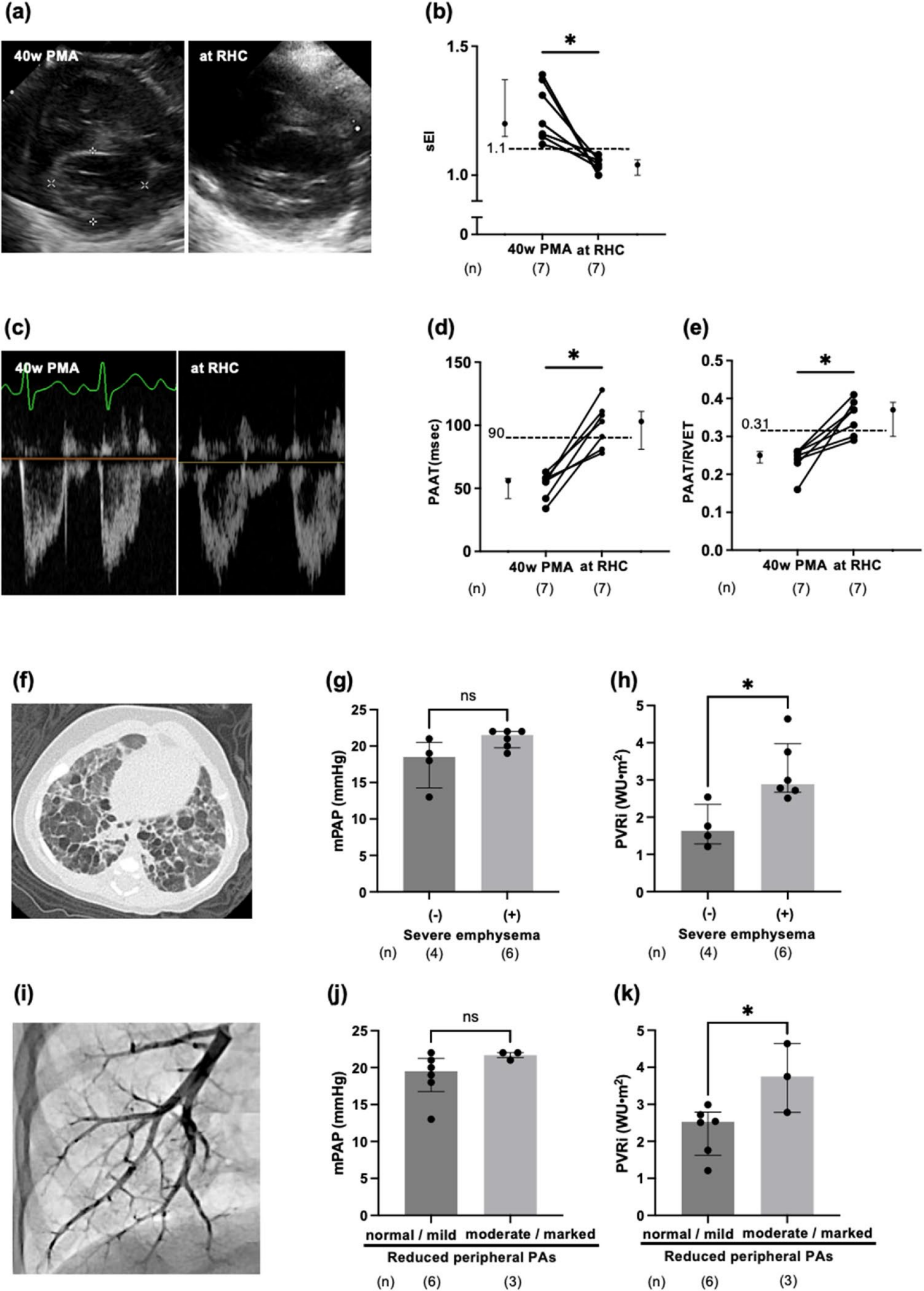
Changes in echocardiographic findings during follow-up and association of pulmonary hemodynamics with chest CT and pulmonary artery wedge angiography findings. **a** Representative echocardiographic findings in a patient (Case 5) showing improvement in systolic eccentricity index (sEI). **b** Changes in sEI from 40 weeks of postmenstrual age (PMA) to the time of right heart catheterization (RHC) (median age of 25 months). **c** Representative echocardiographic findings in a patient (Case 5) showing improvement in pulmonary artery (PA) acceleration time (PAAT) and a ratio of PAAT to right ventricular ejection time (PAAT/RVET). Changes in PAAT (**d**) and PAAT/RVET (**e**) from 40 weeks of PMA to the time of RHC. Dashed-lines indicate the reported cut-off values to indicate the presence of RHC-derived pulmonary hypertension (mean pulmonary artery pressure [mPAP] ≥ 25 mmHg) in the pediatric cohorts. **f** Representative chest CT in a patient (Case 5) showing multiple bullae or blebs and fibrous interstitial shadows. Comparison of mPAP (**g**) and pulmonary vascular resistance index (PVRi) (**h**) in the patients with or without severe emphysema on chest CT. **i** Representative pulmonary wedge angiogram of a patient (Case 5) showing a marked reduction in the contrast filling of the peripheral PAs. Comparison of mPAP (**j**) and PVRi (**k**) in the patients with normal/mild or moderate/marked reduction of contrast filling in peripheral PAs on pulmonary artery wedge angiogram. Numbers in parenthesis indicate the number of the patients. **b**, **d** and **e** Analysis based on data from sildenafil-treated patients only. Individual values are presented with median and IQR. Wilcoxon signed-rank test were used. **g**, **h**, **j** and **k** Analysis with data from all patients. Values are presented as median and IQR. Mann–Whitney *U* test was used for the analysis.^*^P < *0.05*

Among a total of 56 patients, who were born at GA < 28 weeks and screened for PH at 40 weeks of PMA during 2018 to 2020, 10 patients (18%) with BPD-PH were included. Seven patients (70%) were treated with sildenafil. The remaining 3 patients, who had not been treated with sildenafil, had an atrial septal defect (ASD) at 40 weeks of PMA. There were no cases excluded. No patients died in the present cohort. Clinical characteristics are shown in Supplemental Table S1. Ultrasound study at RHC showed improvement in PH as represented by sEI, PAAT and PAAT/RVET (*p* < 0.05, respectively) compared with those at 40 weeks of PMA in patients with sildenafil therapy (Fig. 1, a-e). Changes in these ultrasound findings of all 10 patients showed similar results (Supplemental Fig.S1 and Supplemental Table S2). The percentage of patients meeting ultrasound-defined criteria for PH represented by sEI > 1.1 (*p* < 0.05) and another index, both of PAAT < 90 ms and PAAT/RVET < 0.31 (*p* < 0.05), at the time of RHC was lower than those at 40 weeks of PMA both in the analyses of all patients (Supplemental Table S3) and sildenafil-treated patients only (Supplemental Table S4). The RHC at a median age of 25 (19–32) months (Table 1) showed mPAP of 21 (19–22) mmHg and pulmonary vascular resistance index (PVRi) of 2.63 (1.95–2.94) Wood units (WU)·m^2^. Of note, 5 patients (50%), including a patient (Case 10) with a significant ASD and PH related to pulmonary overflow, had PH according to the definition of PH (mPAP > 20 mmHg) [4]. In the analysis of RHC in sildenafil-treated patients only (*n* = 7), which showed mPAP of 20 (19–22) mmHg and PVRi of 2.72 (2.53–3.37) WU·m^2^, 3 patients (43%) had PH. In the ultrasound study at RHC, sEI and PAAT were comparable between patients with PH and without PH. PAAT/RVET was lower in patients with PH than those without PH (Supplemental Figure S2). The percentage of patients meeting ultrasound-defined criteria for PH represented by sEI > 1.1 (p > 0.99) and both of PAAT < 90 ms and PAAT/RVET < 0.31 (p = 0.143) at the time of RHC was comparable between patients with and without RHC-defined PH in the analyses of all patients (Supplemental Table S5) and sildenafil-treated patients only (Supplemental Table S6).

**Table 1:** Description of hemodynamics and pulmonary wedge angiography of the individual patients

Case	Associated shunts	PAH medications	Age (month)	Right heart catheterization						Reduction of pulmonary vascularity by angiography (grade)
				AOP s/d/m (mmHg)	PAP s/d/m (mmHg)	PAWP (mmHg)	LVEDP (mmHg)	PVRi (WU･m^2^)	Qp/Qs	
1	small PDA	sildenafil	34	82/51/68	25/11/19	8	8	2.99	1	normal
2	small ASD	none	17	74/45/60	26/12/19	7	9	1.50	1.35	NA
3	none	sildenafil	29	73/47/61	27/15/22	11	12	2.72	1	mild
4	none	sildenafil	24	78/53/66	23/13/18	8	10	2.54	1	normal
5	none	sildenafil	35	70/42/55	30/14/22	8	11	4.64	1	marked
6	none	sildenafil	25	72/40/57	26/16/20	11	15	2.51	1	normal
7	spontaneously closed ASD	none	16	70/43/56	28/15/21	9	11	2.78	1	moderate
8	none	sildenafil	33	98/50/72	30/11/22	10	12	3.75	1	marked
9	none	sildenafil	25	75/50/63	19/10/13	6	8	1.76	1	mild
10	ASD	none	6	65/37/51	28/15/21	8	9	1.21	2.89	normal

Patients with severe emphysema on the initial chest CT (Fig. 1, f) had higher PVRi than those without severe emphysema (*p* < 0.05), despite comparable mPAP (Fig. 1, g and h). Pulmonary wedge angiography showed reduced contrast filling of peripheral PAs in 5 patients (mild: *n* = 2; moderate: *n* = 1; markedly: *n* = 2) (Fig. 1, i, Supplemental Figure S3 and Supplemental Movie a and b). Patients with moderate/marked reduction of PA contrast filling had higher PVRi than those with normal/mild reduction (*p* < 0.05), despite similar mPAP (Fig. 1, j and k). Sildenafil therapy was terminated after RHC in 2 of 7 patients because of the normalization of pulmonary hemodynamics. There were neither sildenafil-related adverse events nor deterioration in findings related to PH during the follow-up period.

## Discussion

The major findings in the present study are threefold. Firstly, despite the apparent resolution of echocardiographic indices of PH, half of the patients still exhibited RHC-derived PH in the mid-term, in accordance with the new PH definition [4]. Secondly, 55% of patients showed decreased pulmonary vascularity on angiography, which correlated with increased PVR, suggesting the persistence of potential pulmonary vascular disease (PVD) associated with BPD. Thirdly, the history of severe emphysema at 40 weeks of PMA correlated with increased PVR, which may be at risk for persistent PVD later in infancy. These findings are consistent with the hypothesis that BPD-PH may persist, albeit mild, in the mid-term, which may be of clinical relevance and that such findinigs may be related to the interplay between vascular development and airway formation at BPD diagnosis.

The present study cohort consists of BPD patients in the mid-term who were stable and were followed up for BPD-PH, with or without treatment with pulmonary vasodilators. The favorable clinical course of PH in the present cohort is consistent with recent reports [2, 3]. The present patients had apparent resolution of echocardiography-derived PH at 2.5 years of corrected age compared with the values at 40 weeks of PMA. However, since such ultrasound-derived PH parameters were previously validated in patients with PA pressure of 25 mmHg and over, the clinical relevance of such indices in patients with mPAP of 21–24 mmHg were unknown. Previous reports using RHC focused on sporadic cases with severe PH assessed in early infancy [11, 12]: there were no cohort studies on the pulmonary hemodynamics and vascular abnormalities in consecutive patients by using RHC. The present study showed, for the first time to our knowledge, that 5 out of 10 patients with severe BPD had PH with an mPAP of 21–24 mmHg. Four of the remaining 5 had a relatively high mPAP of 17–20 mmHg. These findings suggest that even after the resolution of echocardiography-derived PH in BPD-PH patients in the mid-term, the majority of such patient population have PH or mildly elevated PA pressure, accompanied by abnormal pulmonary vasculature, indicating that echocardiography is insufficient and RHC may be necessary to follow up this population of patients.

This study has several limitations. Firstly, it is a retrospective study at a single institution with a small sample size. However, it includes consecutive patients from a prospectively maintained database at the only center in the region's tertiary perinatal care area. Given the incidence of extremely preterm births (GA < 28 weeks, ~ 20/year) in this region, the study covers most such cases during the period. Secondly, it is also limited by other retrospective nature of the study, including variability in the timing of RHC and the presence or absence of pharmacological interventions with pulmonary vasodilators. Thirdly, it does not include the course of patients without a PH diagnosis at screening or more severe cases who died before 36 weeks of PMA. Finally, the prognostic significance of mPAP of 21–24 mmHg in the present patients is unknown in the present study.

This study has several implications. Firstly, the persistence of PH and pulmonary vascular hypoplasia in a half of children with a history of BPD-PH warrants multidisciplinary collaborative studies on the long-term prognosis of BPD-PH in childhood and even in adulthood. Secondly, the present study warrants any studies on the assessment of persistent pulmonary vasculopathy (e.g. optimal timing of invasive hemodynamic studies) and pharmacological interventions to prevent PH and pulmonary vasculopathy (e.g. duration and discontinuation criteria for PH-specific treatment) observed in childhood.

In conclusion, the present RHC-based study showed that mild PH associated with impaired pulmonary vascular growth persists at least into childhood in a half of children with a history of BPD-PH, despite apparent resolution of ultrasound-defined PH. This study warrants future prospective studies with larger sample size focusing on the long-term prognosis of BPD-PH and herald developing any diagnostic and therapeutic strategies to prevent cardiopulmonary sequelae associated with BPD.

## Supplementary Information

Below is the link to the electronic supplementary material.

Supplementary file1 (PDF 2217 KB)

Supplementary file2 (MOV 1792 KB)

Supplementary file3 (MOV 937 KB)

## Data Availability

The data that support the findings of this study are included in the article and supplementary information and the further inquiries can be directed to the corresponding authors.

## Abbreviations

AOP: Aortic pressure
ASD: Arterial septal defect
BPD: Bronchopulmonary dysplasia
BPD-PH: Bronchopulmonary dysplasia-associated pulmonary hypertension
CHD: Congenital heart disease
d: Diastolic
GA: Gestational age
IQR: Interquartile range
LVEDP: Left ventricular end-diastolic pressure
m: Mean
mPAP: Mean pulmonary artery pressure
NA: Not applicable
PAAT: Pulmonary artery acceleration time
PAAT/RVET: A ratio of pulmonary artery acceleration time to right ventricular ejection time
PAH: Pulmonary arterial hypertension
PAWP: Pulmonary artery wedge pressure
PDA: Patent ductus arteriosus
PH: Pulmonary hypertension
PMA: Postmenstrual age
PVD: Pulmonary vascular disease
PVRi: Pulmonary vascular resistance index
Qp: Pulmonary blood flow
Qs: Systemic blood flow
RHC: Right heart catheterization
s: Systolic
sEI: Left ventricular end-systolic eccentricity index
WU: Wood units

## References

[1] Abman SH (2001) Bronchopulmonary dysplasia: “a vascular hypothesis.” Am J Respir Crit Care Med 164:1755–1756 11734417 10.1164/ajrccm.164.10.2109111c

[2] Arjaans S, Haarman MG, Roofthooft MTR, Fries MWF, Kooi EMW, Bos AF, Berger RMF (2021) Fate of pulmonary hypertension associated with bronchopulmonary dysplasia beyond 36 weeks postmenstrual age. Arch Dis Child Fetal Neonatal Ed 106:45–50 32571832 10.1136/archdischild-2019-318531 PMC7788204

[3] Altit G, Bhombal S, Hopper RK, Tacy TA, Feinstein J (2019) Death or resolution: the “natural history” of pulmonary hypertension in bronchopulmonary dysplasia. J Perinatol 39:415–425 30617286 10.1038/s41372-018-0303-8

[4] Humbert M, Kovacs G, Hoeper MM, Badagliacca R, Berger RMF, Brida M, Carlsen J et al (2022) 2022 ESC/ERS guidelines for the diagnosis and treatment of pulmonary hypertension. Eur Heart J 43:3618–3731 36017548 10.1093/eurheartj/ehac237

[5] Burkett DA, Patel SS, Mertens L, Friedberg MK, Ivy DD (2020) Relationship Between Left Ventricular Geometry and Invasive Hemodynamics in Pediatric Pulmonary Hypertension. Circ Cardiovasc Imaging 13:e009825 32408829 10.1161/CIRCIMAGING.119.009825 PMC7236425

[6] Levy PT, Patel MD, Groh G, Choudhry S, Murphy J, Holland MR, Hamvas A, Grady MR, Singh GK (2016) Pulmonary Artery Acceleration Time Provides a Reliable Estimate of Invasive Pulmonary Hemodynamics in Children. J Am Soc Echocardiogr 29:1056–1065 27641101 10.1016/j.echo.2016.08.013 PMC5408579

[7] Nagiub M, Kanaan U, Simon D, Guglani L (2017) Risk Factors for Development of Pulmonary Hypertension in Infants with Bronchopulmonary Dysplasia: Systematic Review and Meta-Analysis. Paediatr Respir Rev 23:27–32 28188008 10.1016/j.prrv.2016.11.003

[8] Jobe AH, Bancalari E (2001) Bronchopulmonary dysplasia. Am J Respir Crit Care Med 163:1723–1729 11401896 10.1164/ajrccm.163.7.2011060

[9] Ochiai M, Hikino S, Yabuuchi H, Nakayama H, Sato K, Ohga S, Hara T (2008) A new scoring system for computed tomography of the chest for assessing the clinical status of bronchopulmonary dysplasia. J Pediatr 152:90-95. e3 18154907 10.1016/j.jpeds.2007.05.043

[10] Rabinovitch M, Keane JF, Fellows KE, Castaneda AR, Reid L (1981) Quantitative Analysis of the Pulmonary Wedge Angiogram in Congenital Heart Defects. Circulation 63:152–164 7470217 10.1161/01.cir.63.1.152

[11] del Cerro MJ, Sabate Rotes A, Carton A, Deiros L, Bret M, Cordeiro M, Verdu C, Barrios MI, Albajara L, Gutierrez-Larraya F (2014) Pulmonary hypertension in bronchopulmonary dysplasia: clinical findings, cardiovascular anomalies and outcomes. Pediatr Pulmonol 49:49–59 23788443 10.1002/ppul.22797

[12] Steurer MA, Nawaytou H, Guslits E, Colglazier E, Teitel D, Fineman JR, Keller RL (2019) Mortality in infants with bronchopulmonary dysplasia: Data from cardiac catheterization. Pediatr Pulmonol 54:804–813 30938937 10.1002/ppul.24297

