# Supplementary material for: Persistent pulmonary hypertension in children after apparent resolution of ultrasound-defined pulmonary hypertension associated with bronchopulmonary dysplasia
Source: Eur J Pediatr. 2024 Nov 19;184(1):26. doi: 10.1007/s00431-024-05843-6 (PMC11573848; doi:10.1007/s00431-024-05843-6)
Supplement: Supplementary file 1 — Supplementary file1 (PDF 2217 KB) [file 431_2024_5843_MOESM1_ESM.pdf]

Online Supplementary Information

Persistent Pulmonary Hypertension in Children after Apparent Resolution of Ultrasound-Defined Pulmonary Hypertension Associated with Bronchopulmonary Dysplasia

Mami Takeoka MD, Hirofumi Sawada MD, PhD, Yoshihide Mitani MD, PhD, Hiroyuki Ohashi MD, Noriko Yodoya MD, PhD, Kazunobu Ohya MD, Naoki Tsuboya MD, Tomoya Harada MD and Masahiro Hirayama MD, PhD

Affiliation: Department of Pediatrics, Mie University Graduate School of Medicine, 2-174 Edobashi Tsu Mie, Japan 514-8507

Correspondence

Hirofumi Sawada, MD PhD, E-mail: hisawada@med.mie-u.ac.jp

Yoshihide Mitani, MD PhD, E-mail: ymitani@med.mie-u.ac.jp

Table of contents

Supplemental Table S1: Description of clinical data of the individual patients

Supplemental Table S2: The echocardiographic findings of the patients

Supplemental Table S3: Changes of proportion of the patients who meet the echocardiographic criteria for pulmonary hypertension between 40 weeks of postmenstrual age and at right heart catheterization. (Analysis based on data from all patients)

Supplemental Table S4: Changes of proportion of the patients who meet the echocardiographic criteria for pulmonary hypertension between 40 weeks of postmenstrual age and at right heart catheterization. (Analysis based on data from sildenafil-treated patients only)

Supplemental Table S5: Comparison of proportion of the patients who meet the echocardiographic criteria for pulmonary hypertension between patients with mean pulmonary artery pressure of ≤20 mmHg and of >20 mmHg at right heart catheterization. (Analysis based on data from all patients)

Supplemental Table S6: Comparison of proportion of the patients who meet the echocardiographic criteria for pulmonary hypertension between patients with mean pulmonary artery pressure of ≤20 mmHg and of >20 mmHg at right heart catheterization. (Analysis based on data from sildenafil-treated patients only)

Supplemental Figure S1: Comparison of echocardiographic findings between 40 weeks of postmenstrual age and at right heart catheterization (Analysis based on data from all patients)

Supplemental Figure S2: Comparison of echocardiographic findings at right heart catheterization between patients with mean pulmonary artery pressure of ≤20 mmHg and of >20 mmHg

Supplemental Figure S3: Pulmonary artery wedge angiogram

Supplemental Movie Caption: Pulmonary artery wedge angiogram

Abbreviations

AGA = appropriate for gestational age; ASD = arterial septal defect; BW = body weight; BPD = bronchopulmonary dysplasia; CAM = chorioamnionitis; d=diastolic; GA = gestational age; HFD = heavy-for-date; IQR = interquartile range; m=mean; mPAP = mean pulmonary artery pressure; NA=Not applicable; PAAT = pulmonary artery acceleration time; PAAT/RVET = a ratio of pulmonary artery acceleration time to right ventricular ejection time; PDA = patent ductus arteriosus; PMA = postmenstrual age; PROM = premature rupture of membrane; RHC = right heart catheterization; s = systolic; sEI = left ventricular end-systolic eccentricity index; SGA = small for gestational age; TR= tricuspid regurgitation

Supplemental Table S1 Description of clinical data of the individual patients

Case	GA (wk)	BW (g)	Sex	Proportion at birth	Maternal complications			Ventilation duration (day)	BPD severity (grade)	Chest CT at 40 weeks of PMA		
					PROM	CAM	Oligohydramnios			Hyperexpansion	Multiple bullae or blebs	Fibrous/interstitial abnormalities
1	22 5/7	544	F	AGA	+	+	−	68	severe	+	+	+
2	22 6/7	528	F	AGA	−	+	−	62	mild	+	−	+
3	23 2/7	612	F	AGA	+	+	+	59	severe	+	+	+
4	23 5/7	563	M	AGA	+	+	−	60	severe	−	−	+
5	24 0/7	687	M	AGA	+	+	+	68	severe	+	+	+
6	24 4/7	595	F	AGA	−	−	+	50	severe	+	+	+
7	25 4/7	956	M	HFD	−	+	−	33	severe	+	+	+
8	25 6/7	588	M	SGA	+	+	+	40	severe	+	+	+
9	26 1/7	927	M	AGA	−	+	−	64	severe	+	−	+
10	27 5/7	783	F	SGA	+	−	−	28	severe	+	−	+

Supplemental Table S2 The echocardiographic findings of the patients

Case	Associated shunts	40 weeks of PMA				at RHC				PAP s/d/m (mmHg)
		TR	sEI	PAAT	PAAT/RVET	TR	sEI	PAAT	PAAT/RVET	
1	small PDA	trivial	1.20	58	0.23	trivial	1.04	111	0.37	25/11/19
2	small ASD	trivial	1.16	69	0.31	trivial	1.04	NA	NA	26/12/19
3	none	trivial	1.12	56	0.25	trivial	1.06	81	0.30	27/15/22
4	none	trivial	1.16	34	0.16	trivial	1.08	91	0.39	23/13/18
5	none	trivial	1.31	42	0.26	trivial	1.06	108	0.33	30/14/22
6	none	trivial	1.37	55	0.26	trivial	1.00	128	0.41	26/16/20
7	spontaneously closed ASD	trivial	1.11	61	0.28	trivial	1.06	78	0.30	28/15/21
8	none	trivial	1.39	58	0.24	trivial	1.00	78	0.29	30/11/22
9	none	3.0m/s	1.15	63	0.26	2.3m/s	1.03	103	0.37	19/10/13
10	ASD	trivial	1.33	56	0.28	trivial	1.40	NA	NA	28/15/21

Supplemental Table S3

Changes of proportion of the patients who meet the echocardiographic criteria for pulmonary hypertension between 40 weeks of postmenstrual age and at right heart catheterization.

(Analysis based on data from all patients)

	40wks PMA (n=10)	at RHC (n=10)	P value
Number of patients: sEI >1.1, (%)	10, (100)	1, (10)	p <0.0001
(Number of patients with available data)	(10)	(10)	
Number of patients: PAAT <90 msec and PAAT/RVET <0.31, (%)	9, (90)	3, (38)	p =0.043
(Number of patients with available data)	(10)	(8)	

Supplemental Table S4

Changes of proportion of the patients who meet the echocardiographic criteria for pulmonary hypertension between 40 weeks of postmenstrual age and at right heart catheterization.

(Analysis based on data from sildenafil-treated patients only)

	40wks PMA (n=7)	at RHC (n=7)	P value
Number of patients: sEI >1.1, (%)	7, (100)	0, (0)	p <0.0006
(Number of patients with available data)	(7)	(7)	
Number of patients: PAAT <90 msec and PAAT/RVET <0.31, (%)	7, (100)	2, (29)	p =0.021
(Number of patients with available data)	(7)	(7)	

Supplemental Table S5

Comparison of proportion of the patients who meet the echocardiographic criteria for pulmonary hypertension between patients with mean pulmonary artery pressure of ≤20 mmHg and of >20 mmHg at right heart catheterization.

(Analysis based on data from all patients)

	mPAP ≤20mmHg (n=5)	mPAP >20 mmHg (n=5)	P value
Number of patients: sEI >1.1, (%)	0, (0)	1, (20)	p >0.99
(Number of patients with available data)	(5)	(5)	
Number of patients: PAAT <90 msec and PAAT/RVET <0.31, (%)	0, (0)	3, (75)	p =0.143
(Number of patients with available data)	(4)	(4)	

Supplemental Table S6

Comparison of proportion of the patients who meet the echocardiographic criteria for pulmonary hypertension between patients with mean pulmonary artery pressure of ≤20 mmHg and of >20 mmHg at right heart catheterization.

(Analysis based on data from sildenafil-treated patients only)

	mPAP ≤20mmHg (n=4)	mPAP >20 mmHg (n=3)	P value
Number of patients: sEI >1.1, (%)	0, (0)	0, (0)	p >0.99
(Number of patients with available data)	(4)	(3)	
Number of patients: PAAT <90 msec and PAAT/RVET <0.31, (%)	0, (0)	2, (67)	p =0.143
(Number of patients with available data)	(4)	(3)	

Supplemental Figure S1

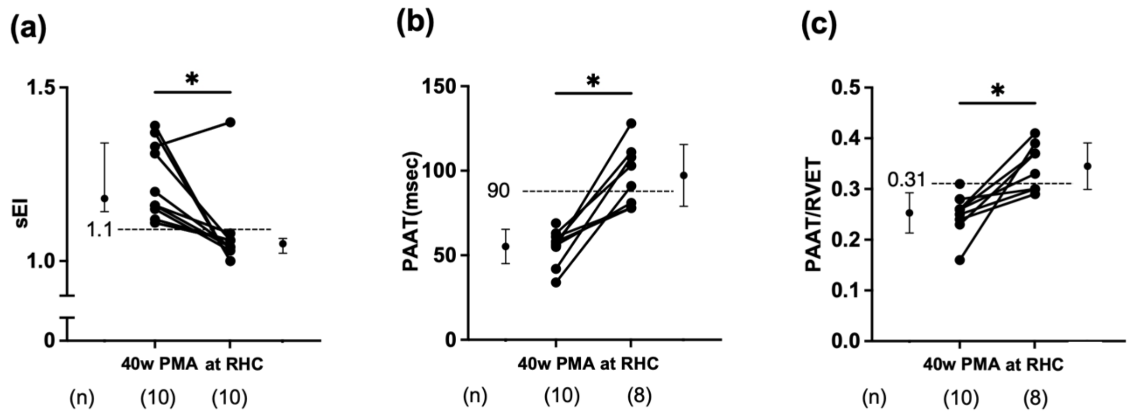

Supplemental Figure S1

Comparison of echocardiographic findings between 40 weeks of postmenstrual age and at right heart catheterization. (Analysis based on data from all patients)

Changes in left ventricular end-systolic eccentricity index (sEI) (a), pulmonary artery acceleration time (PAAT) (b) and a ratio of PAAT to right ventricular ejection time (PAAT/RVET) (c) from 40 weeks of postmenstrual age to the time of right heart catheterization. Individual values are presented with median and IQR for sEI or mean ± SD for PAAT and PAAT/RVET. Wilcoxon signed-rank test for sEI and paired *t*-test for PAAT and PAAT/RVET were used. $^{*}P <0.05.$

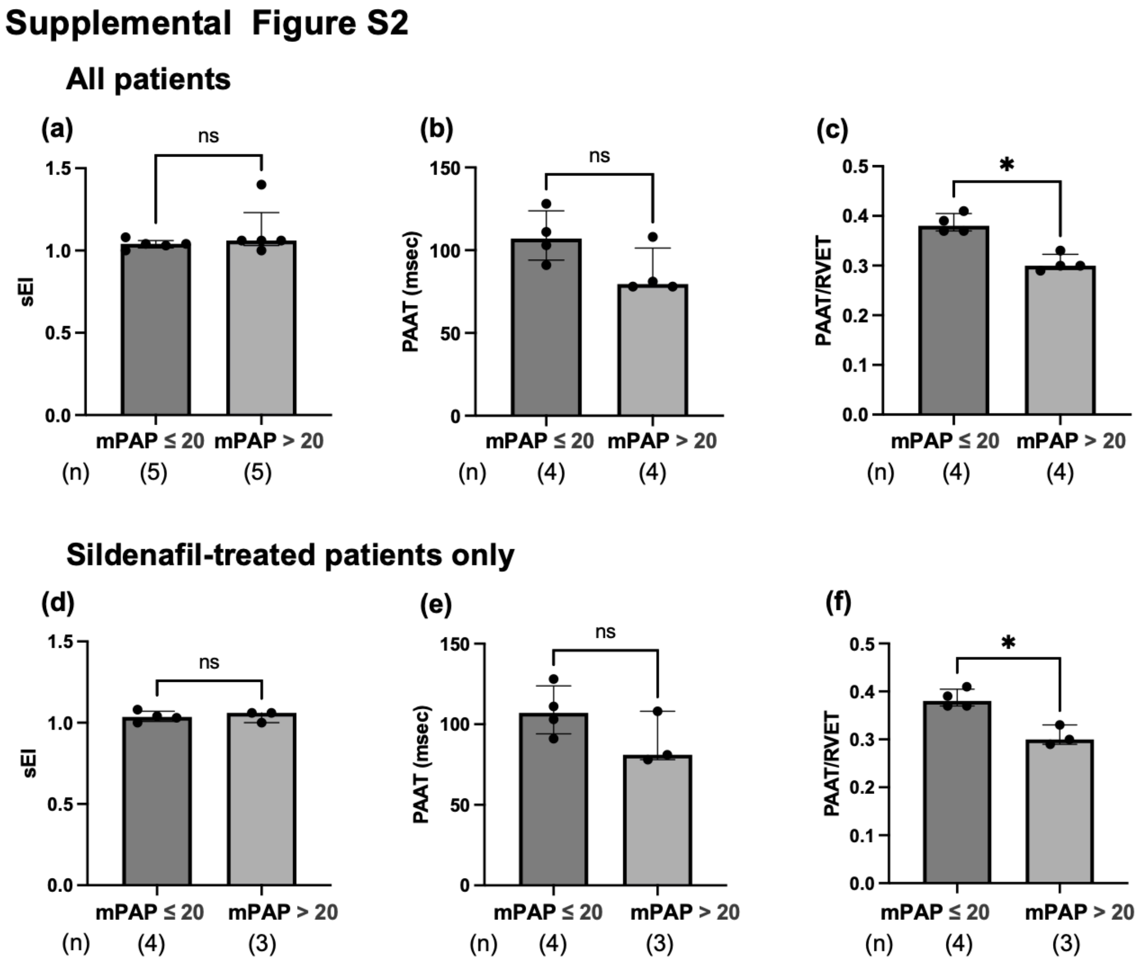

Supplemental Figure S2

Comparison of echocardiographic findings at right heart catheterization between patients with mean pulmonary artery pressure of ≤20 mmHg and of >20 mmHg.

All patient data were included in (a) (b) (c). Only patients with sildenafil therapy were included in (d) (e) (f). Echocardiographic findings at right heart catheterization were not significantly different in left ventricular end-systolic eccentricity index and pulmonary artery acceleration time (PAAT) compared with and without pulmonary hypertension. A ratio of PAAT to right ventricular ejection time was lower in patients with mean pulmonary artery pressure >20 mmHg, both in all patients and in those treated with sildenafil.

Numbers in parenthesis indicate the number of the patients with available data. Values are presented as median and IQR. Mann-Whitney *U* test was used for the analysis. $^{*}P < 0.05$.

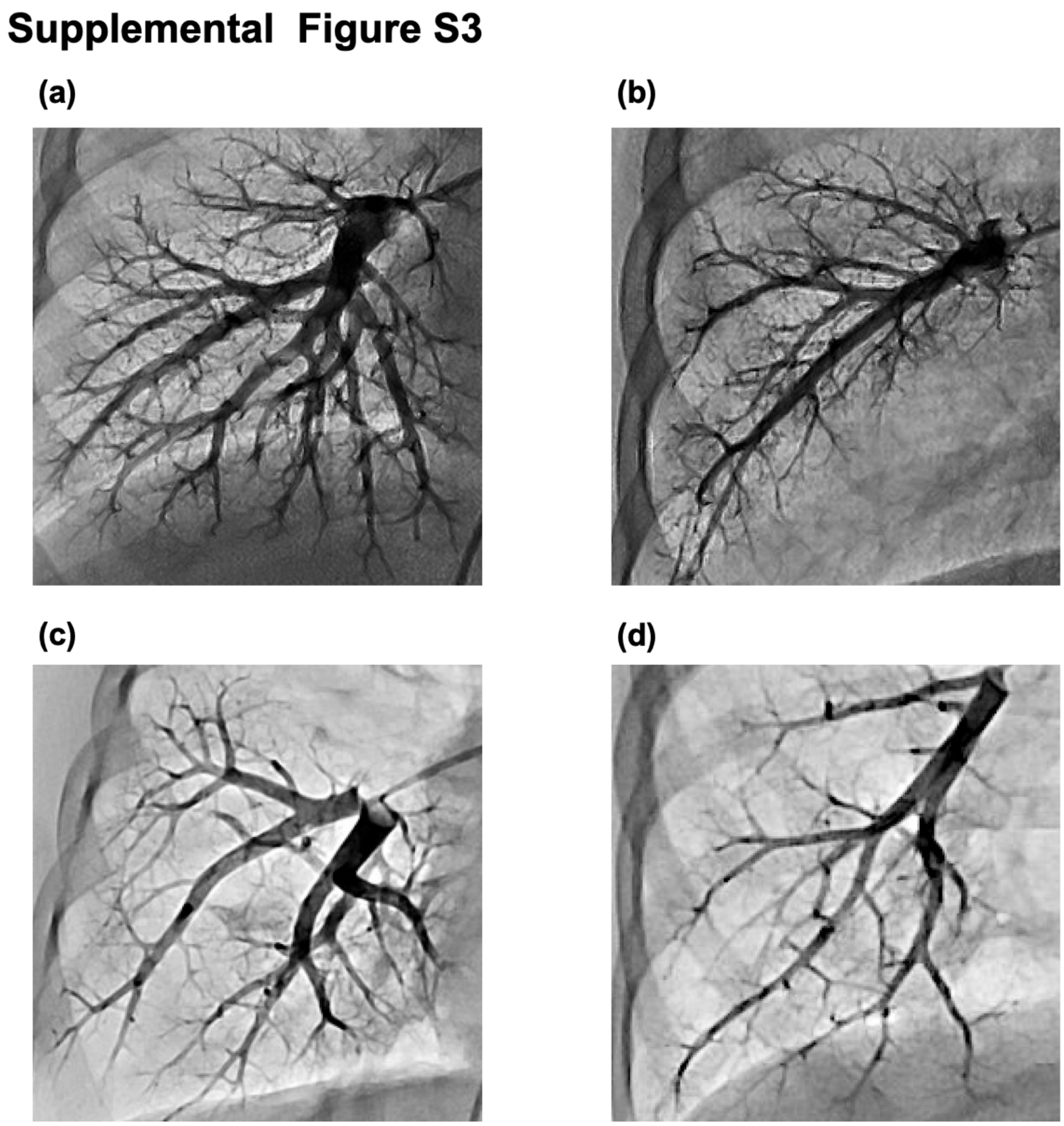

Supplemental Figure S3

Pulmonary artery wedge angiogram

Representative pulmonary wedge angiogram of a patient showing normal (a, Case 4), mild (b, Case 9), moderate (c, Case 7), marked (d, Case 5) reduction in contrast filling of the peripheral pulmonary arteries.

Supplemental Movie

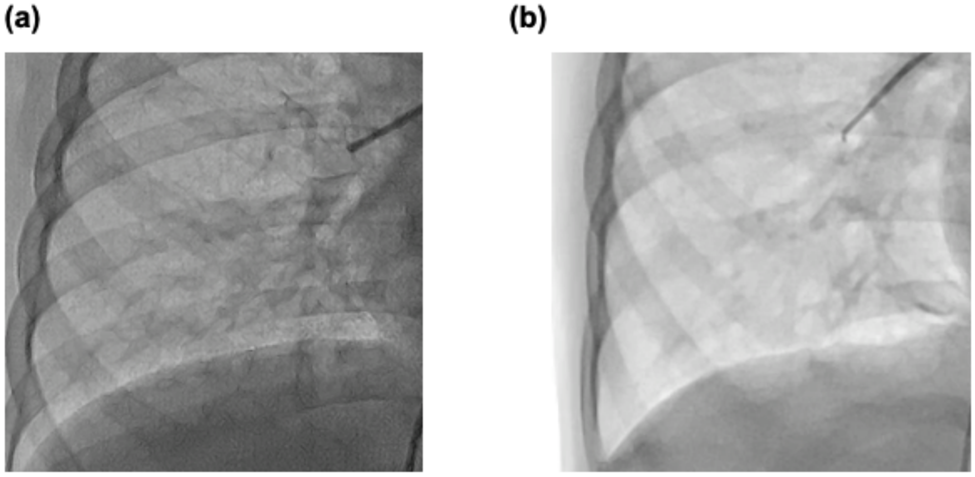

Movie files for these images are uploaded as separate files, supplemental movie (a) and (b).

Supplemental Movie

Pulmonary artery wedge angiogram

Representative movies of pulmonary artery wedge angiograms of patients showing normal (a, Case 4) or markedly (b, Case 5) reduced contrast filling in the peripheral pulmonary artery.
